# Absence of rearrangements in the *BRCA2* gene in human cancers

**DOI:** 10.1054/bjoc.2000.1577

**Published:** 2001-01

**Authors:** S-F Chin, Q Wang, A Puisieux, C Caldas

**Affiliations:** CRC Department of Oncology, University of Cambridge and Cambridge Institute for Medical Research/Wellcome Trust Centre for Molecular Mechanisms in Disease, Box 139, Addenbrookes Hospital, Hills Road, Cambridge, CB2 2XY, UK; Unite d'Oncologie Moleculaire, Unite INSERM U453, Centre Leon Berard, Lyon Cedex 08, 69373, France

**Keywords:** *BRCA2*, southern blot, human cancers, rearrangement, deletion

## Abstract

Mutations of *BRCA2* in sporadic breast and ovarian carcinomas are exceedingly rare. This led to the suggestion that large genomic rearrangements could be involved. We performed Southern blots in genomic DNA from 130 primary breast cancers and 83 cancer cell lines (breast, ovarian, pancreatic and small cell lung carcinomas) and found no genomic rearrangements. These results suggest that a gene other than *BRCA2* is the target of the frequent 13q12.3 allelic deletions in human cancers. © 2001 Cancer Research Campaign http://www.bjcancer.com


*British Journal of Cancer* (2001) **84**(2), 193–195
© 2001 Cancer Research Campaign
doi: 10.1054/ bjoc.2000.1577, available online at http://www.idealibrary.com on IDEAL®

http://www.bjcancer.com

Short Communication

### Absence of rearrangements in the *BRCA2* gene in human cancers

**S-F Chin[1], Q Wang[2], A Puisieux[2] and C Caldas[1]**

[1]CRC Department of Oncology, University of Cambridge and Cambridge Institute for Medical Research/Wellcome Trust Centre for Molecular Mechanisms in Disease, Box 139, Addenbrookes Hospital, Hills Road, CB2 2XY, Cambridge, UK; [2]Unite d'Oncologie Moleculaire, Unite INSERM U453, Centre Leon Berard, 69373 Lyon Cedex 08, France

**Summary** Mutations of *BRCA2* in sporadic breast and ovarian carcinomas are exceedingly rare. This led to the suggestion that large genomic rearrangements could be involved. We performed Southern blots in genomic DNA from 130 primary breast cancers and 83 cancer cell lines (breast, ovarian, pancreatic and small cell lung carcinomas) and found no genomic rearrangements. These results suggest that a gene other than *BRCA2* is the target of the frequent 13q12.3 allelic deletions in human cancers. © 2001 Cancer Research Campaign http://www.bjcancer.com

**Keywords**: *BRCA2*; southern blot; human cancers; rearrangement; deletion

*BRCA2*, the second hereditary breast cancer gene, has been mapped to chromosome 13q12–13 (Wooster et al, 1994). *BRCA2* is a very large gene spanning more than 70 kb of genomic DNA encoding 3495 amino acids (Wooster et al, 1995; Tavtigian et al, 1996). To date more than 300 distinct germline mutations of *BRCA2* (http://www.nhgri.nih.gov/intramural_ research/lab_transfers/bic) have been identified that predispose carriers to breast cancer and to a lesser extent ovarian cancer (Rahman and Stratton, 1998). A small increase in risk for pancreatic and prostate cancer has also been reported in *BRCA2* pedigrees (Wooster et al, 1995; Lancaster et al, 1996; Phelan et al, 1996). Frequent loss of heterozygosity (LOH) at the *BRCA2* locus in a variety of sporadic cancers e.g. breast, ovarian (Lancaster et al, 1996), pancreatic, prostate (Cooney et al, 1996; Li et al, 1998), hepatocellular cancer (Kuroki et al, 1995), suggests this gene may behave as a tumour suppressor gene (Cleton-Jansen et al, 1995). However, no clear disease-causing somatic mutations have been described in *BRCA2* in sporadic breast cancers, and somatic mutations in ovarian cancers are very rare (Foster et al, 1996; Lancaster et al, 1996; Miki et al, 1996; Takahashi et al, 1996; Teng et al, 1996). The lack of somatic *BRCA2* mutations in sporadic breast and ovarian cancers could be due to the mutation detection assays used. Mutations may be missed if they are outside of the region of analysis and certain types of mutations (large deletions, insertions and duplications) may not be detected by PCR-based mutation detection assays. Southern blot analyses have identified 5 large Alu-mediated genomic deletions (Petrij-Bosch et al, 1997; Puget et al, 1997b; Swensen et al, 1997) and a 6 kb Alu-mediated duplication (Puget et al, 1997a) involving *BRCA1* in breast cancer families that would have been missed by conventional PCR-based mutation screening methods such as SSCP, PTT or direct sequencing using genomic DNA as template. A recent study found one case of sporadic breast cancer out of 81 studied with *BRCA1* genomic deletions (van der Looij et al, 2000). Similar large genomic deletions have also been described in other tumour suppressor genes e.g. *p53* (Masuda et al, 1987; Ruggeri et al, 1992), *hMLH1* (Nystrom-Lahti et al, 1995), *hMSH2* (Wijnen et al, 1998) and *Rb-1* (Ruggeri et al, 1992). Thus, we wanted to investigate whether similar genomic deletions occur in *BRCA2* which may have escaped detection using PCR-based techniques.

*Received 4 July 2000*
*Revised 4 October 2000*
*Accepted 18 October 2000*

*Correspondence to:* C Caldas

### MATERIALS AND METHODS

We undertook Southern blot analysis of genomic DNA in a large series of 130 invasive breast tumours comprising ductal, lobular, mucinous, tubular, cribriform and squamous cell metaplastic carcinomas. These tumours were snap frozen at the point of collection. Genomic DNA from these primary tumours was digested with EcoRI. In addition, genomic DNA from established cell lines derived from breast (39), ovarian (29), pancreatic (7) and small cell lung (SCLC) tumours (8) was digested with 4 different restriction endonucleases (BamHI, EcoRI, HindIII and PstI). Digested DNA was size fractionated by electrophoresis and transferred onto nylon membranes. Filters were hybridized separately with two clones containing *BRCA2* cDNA. The first is a 5.8 kb fragment representing amino acids 1–1963 (*BRCA2*-front) and the second fragment is 4.6 kb from amino acids 1895–3495 (*BRCA2*-back).

### RESULTS

Initially, we detected aberrant-sized fragments in two primary tumours; tumour 386 with both probes and tumour 64 NT with *BRCA2*-front probe (Figure 1). There were 8 breast cancer cell lines that showed restriction fragment size fragment abnormalities with *BRCA2*-front and 4 with *BRCA2*-back. In ovarian cancer cell lines, abnormalities were only observed in 3 cell lines using *BRCA2*-front. No abnormalities were detected in any of the SCLC or pancreatic cell lines. A representative sample of these Southern blot experiments is presented in Figure 1.

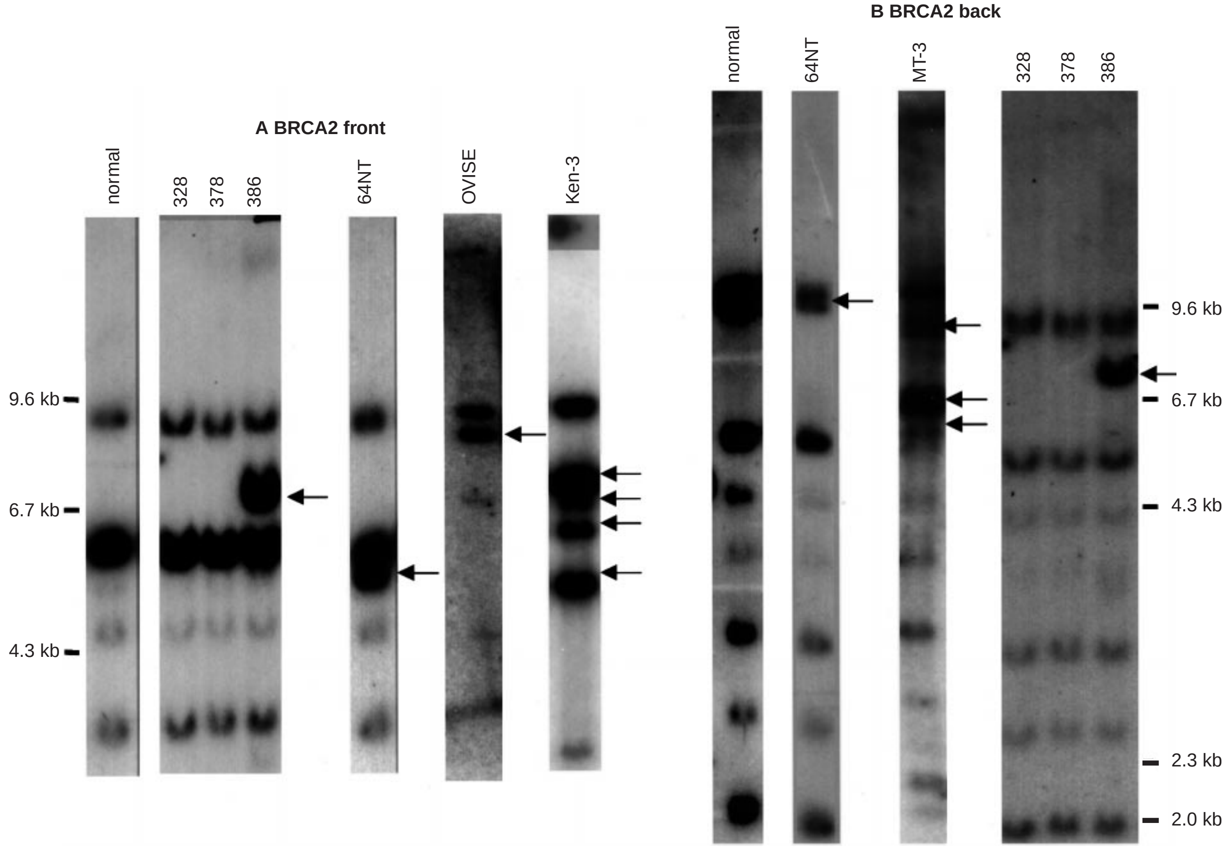

**Figure 1** Southern analysis of *BRCA2* gene in primary breast tumours (328, 378, 386, 64NT), breast cancer cell line (MT-3), and ovarian cancer cell lines (OVISE, KEN-3) digested with EcoRI. Hybridization with (**A**) *BRCA2*-front and (**B**) *BRCA2*-back shows aberrant restriction fragments

To confirm the presence of genomic rearrangements in the abnormal samples, the experiments were repeated with longer incubation of the DNA with the respective restriction endonucleases to ensure complete digestion. No abnormal restriction fragments were detected in any of the samples suggesting that the initial abnormal bands were due to incomplete digestion (data not shown). While no large genomic deletions were observed in the *BRCA2* gene, restriction fragment length polymorphisms were observed in both primary tumours and cell lines (Figure 2).

### DISCUSSION

In summary, *BRCA2* does not undergo large intragenic deletions in human tumours. Only 8 somatic *BRCA2* mutations, 3 in breast tumours (Lancaster et al, 1996; Miki et al, 1996; Weber et al, 1996), 4 in ovarian cancers (Foster et al, 1996; Takahashi et al, 1996) and one in a hepatocellular carcinoma (Katagiri et al, 1996), have been reported since the discovery of the gene using PCR-based mutation detection assays. Similar to *BRCA1*, the region containing *BRCA2* undergoes loss of heterozygosity in a fraction of breast and ovarian tumours (Cleton-Jansen et al, 1995). In fact in another study using these cell lines, we found that 24/36 breast (67%), 6/30 ovarian (20%), 4/7 pancreatic (57%) and 5/8 SCLC (63%) used in this study had homozygosity for all markers tested in the region encompassing *BRCA2* (data not shown) suggestive of allelic deletions. Partial mutational analyis of *BRCA2* mutations was undertaken for some of the cell lines with LOH and to date, only one breast cancer cell line with LOH, MT-3, was found to have a 1 bp deletion in exon 23 of *BRCA2* (KL Gorringe & C Caldas, unpublished data). Like *BRCA1*, the lack of *BRCA2* mutations in sporadic breast and ovarian cancers

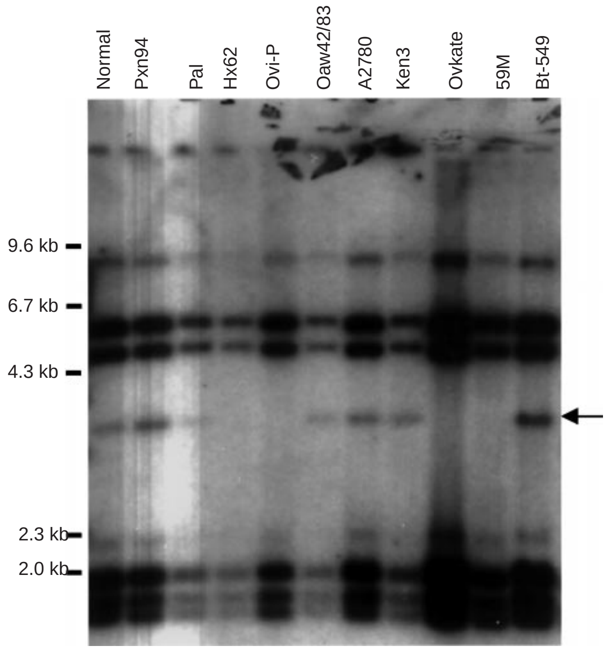

**Figure 2** Restriction fragment length polymorphism seen in the normal control, breast (BT-549) and ovarian cancer cell lines digested with Pst-1 and hybridized with *BRCA2*-front

*© 2001 Cancer Research Campaign*

suggest either distinct genetic pathways or different mechanisms for inactivating gene function compared to the familial forms (Rahman and Stratton, 1998). The high frequency of LOH on chromosome 13q could be explained by the close proximity of other tumour suppressor genes e.g. retinoblastoma (Lee et al, 1988), Brush-1 (Schott et al, 1994) or other putative tumour suppressor gene(s) that might be targeted instead of *BRCA2*.

### ACKNOWLEDGEMENTS

This research is supported by the Cancer Research Campaign (CRC) and Ligue contre le Cancer. We thank Dr Yasuhiko Kiyozuka for the KEN-3 cell line, Dr Itsuo Gorai for the OVISE and OVKATE cell lines and Dr Mike Bibby for the MT-3 cell line. The *BRCA2* cDNA clones were a kind gift from Dr Tony Kouzarides.

### REFERENCES

Cleton-Jansen AM, Collins N, Lakhani SR, Weissenbach J, Devilee P, Cornelisse CJ and Stratton MR (1995) Loss of heterozygosity in sporadic breast tumours at the BRCA2 locus on chromosome 13q12-q13. *Br J Cancer* **72**: 1241–1244

Cooney KA, Wetzel JC, Merajver SD, Macoska JA, Singleton TP and Wojno KJ (1996) Distinct regions of allelic loss on 13q in prostate cancer. *Cancer Research* **56**: 1142–1145

Foster KA, Harrington P, Kerr J, Russell P, DiCiccio RA, Scott IV, Jacobs I, Chevenix-Trench G, Ponder BAJ and Gayther SA (1996) Somatic and germline mutations of the BRCA2 gene in sporadic ovarian cancer. *Cancer Research* **56**: 3622–3625

Katagiri T, Nakamura Y and Miki Y (1996) Mutations in the BRCA2 gene in hepatocellular carcinoma. *Cancer Res* **56**: 4575–4577

Kuroki T, Fujiwara Y, Nakamori S, Imaoka S, Kanematsu T and Nakamura Y (1995) Evidence for the presence of two tumour-suppressor genes for hepatocellular carcinoma on chromosome 13q. *Br J Cancer* **72**: 383–385

Lancaster JM, Wooster R, Mangion J, Phelan CM, Coehran C, Grumbs C, Seal S, Barfoot R, Collins N, Bignell G, Patel S, Hamoudi R, Larsson C, Wiseman RW, Berchuck A, Iglehart JD, Marks JR, Ashworth A, Stratton MR and Futreal PA (1996) BRCA2 mutations in primary breast and ovarian cancers. *Nature Genetics* **13**: 238–240

Lee, EH, To H, Shew JY, Bookstein R, Scully P and Lee WH (1988) Inactivation of the retinoblastoma susceptibility gene in human breast cancers. *Science* **241**: 218–221

Li C, Larsson C, Futreal A, Lancaster J, Phelan C, Aspenblad U, Sundelin B, Liu Y, Ekman P, Auer G and Bergerheim US (1998) Identification of two distinct deleted regions on chromosome 13 in prostate cancer. *Oncogene* **16**: 481–487

Masuda H, Miller C, Koeffler HP, Battifora H and Cline MJ (1987) Rearrangements of the p53 gene in human osteogenic sarcomas. *Proc Natl Acad Sci* **84**: 7716–7719

Miki Y, Katagiri T, Kasumi F, Yoshimoto T and Nakamura Y (1996) Mutation analysis in the BRCA2 gene in primary breast cancers. *Nature Gen* **13**: 245–247

Nystrom-Lahti M, Kristo P, Nicolaides NC, Chang S-Y, Aaltonen LA, Moisio A-L, Jarvinen HJ, Mecklin JK, Kinzler KW, Vogelstein B, de La Chapelle A and Peltomaki P (1995) Founding mutations and Alu-mediated recombination in hereditary colon cancer. *Nature Med* **1**: 1203–1206

Petrij-Bosch A, Peelen T, van Vliet M, van Eijk R, Olmer R, Drusedau M, Hogervorst FBL, Hageman S, Arts PJW, Ligtenberg MJL, Meijers-Heijboer H, Klijn JGM, Vasen HFA, Cornelisse CJ, van't Veer LJ, Bakker E, van Ommen G-JB and Devilee P (1997) BRCA1 genomic deletions are major founder mutations in Dutch breast cancer patients. *Nature Genetics* **17**: 341–345

Phelan CM, Lancaster JM, Tonin P, Gumbs C, Cochran C, Carter R, Ghadirian P, Perret C, Moslehi R, Dion F, Faucher MC, Dole K, Karimi S, Foulkes W, Lounis H, Warner E, Goss P, Anderson D, Larsson C, Narod SA and Futreal PA (1996) Mutation analysis of the BRCA2 gene in 49 site-specific breast cancer families [published erratum appears in Nat Genet 1996 Jul; 13(3):374]. *Nat Genet* **13**: 120–2

Puget N, Serona-Sinilnikova, OM, Stoppa-Lyonnet D, Audoynaud C, Pages S, Lynch HT, Goldgar D, Lenoir GM and Mazoyer S (1997a) An Alu-mediated 6-kb duplication in the *BRCA1* gene: A new founder mutation? *Am J Hum Gen* **64**: 300–302

Puget N, Torchard D, Serona-Sinilnikova OM, Lynch HT, Feunteun J, Lenoir GM and Mazoyer S (1997b) A 1-kb Alu-mediated germline deletion removing BRCA1 exon 17. *Cancer Res* **57**: 828–831

Rahman N and Stratton MR (1998) The genetics of breast cancer susceptibility. *Annual Review of Genetics* **32**: 95–121

Ruggeri B, Zhang S-Y, Caamano J, DiRado M, Flynn SD and Klein-Szanto AJP (1992) Human pancreatic carcinomas and cell lines reveal frequent and multiple alterations in the p53 and Rb-1 tumour suppressor genes. *Oncogene* **7**: 1503–1511

Schott DR, Chang JN, Deng G, Kurisu W, Kuo W-L, Gray J and Smith HS (1994) A candidate tumour suppressor gene in human breast cancers. *Cancer Res* **54**: 1393–1396

Swensen J, Hoffman M, Skolnick MH and Neuhausen SL (1997) Identification of a 14 kb deletion involving the promoter region of *BRCA1* in a breast cancer family. *Hum Mol Gen* **6**: 1513–1517

Takahashi H, Chiu H-C, Bandera CA, Behbakht K, Liu PC, Couch FJ, Weber BL, LiVolsi VA, Furusato M, Rebane BA, Cardonick A, Benjamin I, Morgan MA, King SA, Mikuta JJ, Rubin SC and Boyd J (1996) Mutations of BRCA2 in ovarian carcinomas. *Cancer Res* **56**: 2738–2741

Tavtigian SV, Simard J, Rommens J, Couch F, Shattuck-Eidens D, Neuhausen S, Merajver S, Thorlacius S, Offit K, Stoppa-Lyonnet D, Belanger C, Bell R, Berry S, Bogden R, Chen Q, Davis T, Dumont M, Frye C, Hattier T, Jammulapati S, Janecki T, Jiang P, Kehrer R, Leblanc JF and Goldgar DE (1996) The complete BRCA2 gene and mutations in chromosome 13q-linked kindreds. *Nature Gen* **12**: 333–337

Teng DH-F, Bogden R, Mitchell J, Baumgard M, Bell R, Berry S, Davis T, Ha PC, Kehrer R, Jammulapati S, Chen Q, Offit K, Skolnick MH, Swedlund B, Wong AKC and Kamb A (1996) Low incidence of Brca2 mutations in breast carcinoma and other cancers. *Nature Gen* **13**: 241–244

van der Looij M, Cleton-Jansen A-M, van Eijk R, Morreau H, van Vliet M, Kuipers-Dijkshoorn N, Olah E, Cornelisse CJ and Devilee P (2000) A sporadic breast tumor with somatically acquired complex genomic rearrangement in *BRCA1 Genes Chromosomes Cancer* **27**: 295–302

Weber BHF, Brohm M, Stec I, Backe J and Caffier H (1996) A somatic truncating mutation in BRCA2 in a sporadic breast tumour. *American Journal of Human Genetics* **59**: 962–964

Wijnen J, van der Klift H, Vasen H, Khan PM, Menko F, Tops C, Heijboer HM and Lindhout D (1998) MSH2 genomic deletions are a frequent cause of HNPCC. *Nature Gen* **20**: 326–328

Wooster R, Neuhausen SL, Mangion J, Quirk Y, Ford D, Collins N, Nguyen K, Seal S, Tran T, Averill D, Fields P, Marshall G, Narod S, Lenoir GM, Lynch H, Feunteun J, Devilee P, Cornelisse CJ, Menko FH, Daly PA, Orniston W, McManus R, Pye C, Lewis CM, Cannon-Albright LA, Peto J, Ponder BAJ, Skolnick MH, Easton DF, Goldgar DE and Stratton MR (1994) Localization of a breast cancer susceptibility gene, BRCA2, to chromosome 13q12–13. *Science* **265**: 2088–2090

Wooster R, Bignell G, Lancaster J, Swift S, Seal S, Mangion J, Collins N, Gregory S, Gumbs C, Micklem G, Barfoot R, Hamoudi R, Patel S, Rice C, Biggs P, Hashim Y, Smith A, Connor F, Arason A, Gudmundsson J, Ficenec D, Kelsell D, Ford D, Tonin P, Bishop DT, Spurr NK, Ponder BAJ, Eeles R, Peto J, Devilee P, Cornelisse CJ, Lynch H, Narod S, Lenoir GM, Eglisson V, Barkadottir RB, Easton DF, Bentley DR, Futreal PA, Ashworth A and Stratton MR (1995) Identification of the breast cancer susceptibility gene BRCA2. *Nature* **378**: 789–762

© 2001 Cancer Research Campaign 

